# Sphingosine 1 Phosphate at the Blood Brain Barrier: Can the Modulation of S1P Receptor 1 Influence the Response of Endothelial Cells and Astrocytes to Inflammatory Stimuli?

**DOI:** 10.1371/journal.pone.0133392

**Published:** 2015-07-21

**Authors:** Simona F. Spampinato, Birgit Obermeier, Anne Cotleur, Anna Love, Yukio Takeshita, Yasuteru Sano, Takashi Kanda, Richard M. Ransohoff

**Affiliations:** 1 Neuroinflammation Research Center, Department of Neurosciences, Lerner Research Institute, Cleveland Clinic, Cleveland, Ohio, United States of America; 2 Department of Neurology and Clinical Neuroscience, Yamaguchi University Graduate School of Medicine, Ube, Yamaguchi, Japan; Washington University, UNITED STATES

## Abstract

The ability of the Blood Brain Barrier (BBB) to maintain proper barrier functions, keeping an optimal environment for central nervous system (CNS) activity and regulating leukocytes’ access, can be affected in CNS diseases. Endothelial cells and astrocytes are the principal BBB cellular constituents and their interaction is essential to maintain its function. Both endothelial cells and astrocytes express the receptors for the bioactive sphingolipid S1P. Fingolimod, an immune modulatory drug whose structure is similar to S1P, has been approved for treatment in multiple sclerosis (MS): fingolimod reduces the rate of MS relapses by preventing leukocyte egress from the lymph nodes. Here, we examined the ability of S1P and fingolimod to act on the BBB, using an *in vitro* co-culture model that allowed us to investigate the effects of S1P on endothelial cells, astrocytes, and interactions between the two. Acting selectively on endothelial cells, S1P receptor signaling reduced cell death induced by inflammatory cytokines. When acting on astrocytes, fingolimod treatment induced the release of a factor, granulocyte macrophage colony-stimulating factor (GM-CSF) that reduced the effects of cytokines on endothelium. In an *in vitro* BBB model incorporating shear stress, S1P receptor modulation reduced leukocyte migration across the endothelial barrier, indicating a novel mechanism that might contribute to fingolimod efficacy in MS treatment.

## Introduction

### Blood-Brain Barrier compromise in multiple sclerosis

In the Central Nervous System (CNS) control of the neural microenvironment is essential for proper functioning of the neuronal network. The presence of the Blood Brain Barrier (BBB) preserves an adequate ionic equilibrium for neurotransmission, and controls the access of immune cells [1]. The endothelial barrier is a fundamental component of the BBB and its properties are strictly related to the presence of other cell types and structures (for a comprehensive description of BBB organization and function see [2]). A primary role is played by astrocytes that support neurons, release growth factors and refine neurotransmission. With regard to BBB function it has been demonstrated that specific features of the BBB endothelium are the consequence of its interaction with astrocytes, both through physical contact and soluble factors [3]. A compromised BBB is a hallmark of a number of CNS diseases, such as multiple sclerosis (MS). Associated with active demyelinating multifocal lesions, a leaky BBB can be visualized by magnetic resonance imaging in MS patients and post-mortem evidences indicate focal microvascular leakage [4]. Notably, the BBB is also primarily involved in the pathogenesis of the disease whenever an immune response causes elevated local concentrations of inflammatory cytokines, and thereby induces alterations of the BBB endothelium. For instance the levels of molecules such as selectins, chemokines and adhesion molecules are increased and as consequence, luminal leukocyte-endothelial interactions such as rolling, arrest, and crawling are facilitated. Under these conditions, leukocytes migration across the BBB and infiltration into the CNS is enhanced, perpetuating inflammation, and thus exacerbating the pathology [5].

### Sphingosine 1 phosphate as therapeutic target in MS

To reduce the infiltration of the peripheral blood cells into the CNS several therapeutical approaches have been developed: some affect the interactions between endothelium and circulating leukocytes (e.g. the humanized monoclonal antibody Natalizumab targeting the cell adhesion molecule alpha-4 integrin), while others reduce the egress of leukocytes from lymph nodes into periphery. The latter is the case with fingolimod, a molecule structurally similar to sphingosine-1 phosphate (S1P) [6]. S1P is a bioactive sphingolipid that, acting through its five receptors (S1P_1-5_), modulates a large diversity of biological mechanisms (cell proliferation, survival, cytoskeletal reorganization, and migration). S1P gradients drive egress of leukocytes from lymph nodes [7]. Acting as an S1P_1_ functional antagonist, fingolimod reduces the egress of leukocytes, and in particular, T cells from the lymph node. Fingolimod is now widely used in the treatment of relapsing forms of MS [8]. Although the primary beneficial mechanism of action occurs within lymph nodes, it needs to be considered that S1P receptors are broadly expressed in varied organs [9], indicating that fingolimod may also have effects beyond the reduced release of leukocytes into the periphery. Interestingly S1P exerts important functions towards the endothelium, where it modulates endothelial cell permeability and barrier properties [10, 11]. S1P receptors are also expressed by astrocytes, which proliferate in response to S1P [12], and show enhanced promotion of neuronal survival [13, 14]. Of note the release of S1P and the expression of its receptors are very often modified under pathological conditions, like MS or spinal cord injury [15–17].

We here investigate whether key BBB properties could be modified by S1P receptor modulation, addressing in particular the role exerted by the immunomodulator fingolimod, which is already well-established in the treatment of MS. Using an *in vitro* co-culture system we analyzed the effect of S1P signaling on endothelial cells and astrocytes, two of the principal cellular components of the BBB. We either examined endothelial cells and astrocytes independently or in a more physiological condition in which physical contact between the two cell types was enabled. In the latter case we investigated whether the presence of S1P could modify the ability of immune cells to cross the BBB, using an *in vitro* model incorporating shear stress, that very closely simulates the events that occur during the *in vivo* transmigration of leukocytes into the CNS [18].

The data here reported indicate that S1P biology modulates several pathways both on endothelial cells and astrocytes. S1P rescued endothelial cells from death upon cytokine challenge, either directly, or indirectly through stimulation of astrocytes to release factors. Further, activation of S1P signaling decreased leukocyte transmigration. These findings provide evidence that beneficial effects on the BBB may contribute to the mechanism of action of fingolimod.

## Materials and Methods

### Ethical requirements for human subjects research

The Cleveland Clinic Institutional Review Board approved all study protocols, and signed informed consents were obtained from all blood donors. Healthy volunteers between 20 and 50 years old were recruited. The Cleveland Clinic Institutional Review Board approved all study protocols, and signed informed consents were obtained from all blood donors. Subjects were not experiencing systemic infection or taking nonsteroidal anti-inflammatory drugs (NSAIDs) at the time of phlebotomy.

Human Brain MicroVascular Endothelial Cells (hBMVEC) and human Astrocytes (hAst), adult human immortalized cell lines, were previously described [19, 20].

### Cell culture

Human Brain MicroVascular Endothelial Cells (hBMVEC), an adult human immortalized cell line, are transfected with plasmid expressing temperature sensitive Simian virus-40 large T-antigen (ts-SV40-LT) as previously described [19]. hBMVEC were grown in MCDB-131 media (Sigma-Aldrich) supplemented with EGM-2 SingleQuots (Lonza, Basel Switzerland) and 20% heat-inactivated FBS. Human Astrocytes (hAST) transfected with the same ts-SV40-LT plasmid, as previously described [20], were grown in astrocyte media containing 2% heat-inactivated FBS, astrocyte growth supplement and penicillin/streptomycin solution as provided with the Astrocyte media kit (ScienCell Research Laboratories, Carlsbad, CA). For experiments, both hBMVECs and hAST were grown at 33°C for two days and then transferred to 37°C, where they exhibited growth arrest and differentiation, as previously described [18]. After differentiation for 2 days at 37°C, cells were exposed to cytokines for 24 hours as described below.

### Reagents

Sphingosine-1-phosphate (S1P, Sigma-Aldrich) was dissolved in methanol to a stock concentration of 1 mg/ml. Fingolimod (FTY), NVP-AUY954-AA-11 (AUY), NIBR-0231 (NIRB) were kindly provided by Novartis Pharma (Basel, Switzerland) and dissolved in DMSO to a stock concentration of 10 mM. Fingolimod Phosphate (NVP-AEY366-NX-7, pFTY), also provided by Novartis Pharma, was dissolved in DMSO/HCl to a stock concentration of 10 mM. recombinant human granulocytes and macrophages colony stimulating factor (GM-CSF) and the neutralizing human monoclonal GM-CSF antibody (clone number 3209, purchased by R&D Systems, Minneapolis, MN) were dissolved in sterile PBS. Stock solutions were further diluted in culture media as appropriate before use.

### Assessment of hBMVEC viability

Live/dead cell staining was examined by Trypan blue (Lonza; 0.4% for 5 min) 18 h after exposure to tumor necrosis factor alpha (TNFα 10 U/ml, R&D System) and interferon gamma (IFN γ 5U/ml, R&D Systems). Stained hBMVECs, i.e. dead cells, were manually counted from three to five random fields per well with phase contrast microscopy at a 40x magnification. Each experiment was repeated four times, and every treatment/condition was performed in triplicate.

Cell viability was measured with the MTT colorimetric assay based on the conversion of a diphenyltetrazolium salt into blue formazan by mitochondrial activity of viable cells. After 24 h cytokine exposure, the media was removed and substituted with the MTT solution (0.9 mg/ml; Sigma-Aldrich) for 2 h at 37°C. DMSO was added to allow EC disruption and formazan production was evaluated in a plate reader (absorbance = 570 nm). Each experiment was repeated four times, and every treatment/condition was performed in triplicate.

### GM-CSF production

hAST were exposed for 6 h to TNFα (10 U/ml) and IFNγ (5 U/ml), either alone or in the presence of S1P (50 nM), AUY-954 (50 nM), fingolimod (FTY, 50 nM) or fingolimod phosphate (pFTY, 50 nM). After 6 h the cells were extensively washed and fresh medium was added. 18 h later, the medium was collected and processed either for a Proteome Profiler Human Angiogenesis Array or to Quantikine ELISA Human GM-CSF assay, (both purchased from R&D Systems) according to the manufacturer’s procedure.

### Immunocytochemistry

hBMVEC were exposed for 6 h to cytokines and then fixed. After 0.2% Triton-X permeabilization they were incubated with primary antibody, rabbit anti active caspase-3 (1:100, Abcam) for 5 h at room temperature. F-actin staining (Alexa Fluor 488 Phalloidin, Life Technologies), performed on fixed cells, was executed according to the manufacturer’s procedure.

### PBMC isolation

Peripheral blood mononuclear cells (PBMCs) were isolated from fresh heparinized blood of healthy subjects by density centrifugation with Lymphocyte Separation Medium (Mediatech, Herndon, VA) as previously described [21]. For transmigration assays, PBMCs were resuspended at 10x10^6^ cells in 30 ml of Transendothelial migration buffer, TEM buffer (RPMI 1640 without phenol red + 1% bovine serum albumin + Hepes + L-glutamine + Na-pyruvate + MEM non-essential amino acids) and assayed within 2 h from phlebotomy.

### 3D Flow system and transmigration assay

A 3D flow chamber device (C.B.S. Scientific) was used for transmigration assays as previously described [18]. Briefly, a pump delivers programmable shear flow to a flow chamber that has three reservoirs, each of them enabled to hold a membrane which separates the upper and lower buffer reservoir completely. Flow membranes are 8 mm in diameter, made of tracketched polycarbonate with 3 μm pores, and were coated with rat-tail collagen I solution (50 μg/ml) (BD Bioscience, San Diego, CA). First, 5x10^4^ hAST were seeded on the abluminal side of the membrane, then 12x10^4^ hBMVEC were seeded on the luminal side, and co-cultured in Astrocyte media for 2 days at 33°C. Co-cultures were kept for 2 days at 37°C before any kind of treatment, as indicated. Cells were activated with TNFα (10 U/ml) and IFNγ (5 U/ml) in Astrocyte Media for 24 h at 37°C. CXCL12 (50 ng/ml in TEM buffer, R&D Systems) was applied to the apical hBMVEC layer and incubated for 15 min at 37°C. CCL2 (25 ng/ml in TEM buffer, R&D Systems) was loaded in the bottom wells of the 3D flow chamber and maintained throughout the assay. 10x10^6^ PBMCs (total cells per assay) in 30 ml TEM buffer (kept warm in a 37°C water bath) were perfused via a peristaltic pump through the chamber at a final concentration of 333.000 cells/ml and at a shear stress of 2 dyne/cm^2^ resulting in a total assay time of 60 minutes. All chambers were kept on a 37°C slide warmer for the duration of the assay. Migrated PBMCs were recovered from the bottom chamber and enumerated by a hemocytometer.

### Statistical analysis

All data are expressed as means ± SEM of 3 to 6 different experiments. Data have been analyzed by one-way ANOVA followed by Newmann Keuls test for significance. p<0.05 was taken as the criterion for statistical significance.

## Results

### S1P receptor modulation on endothelial cells modifies their response after cytokine exposure

**Fig 1:**
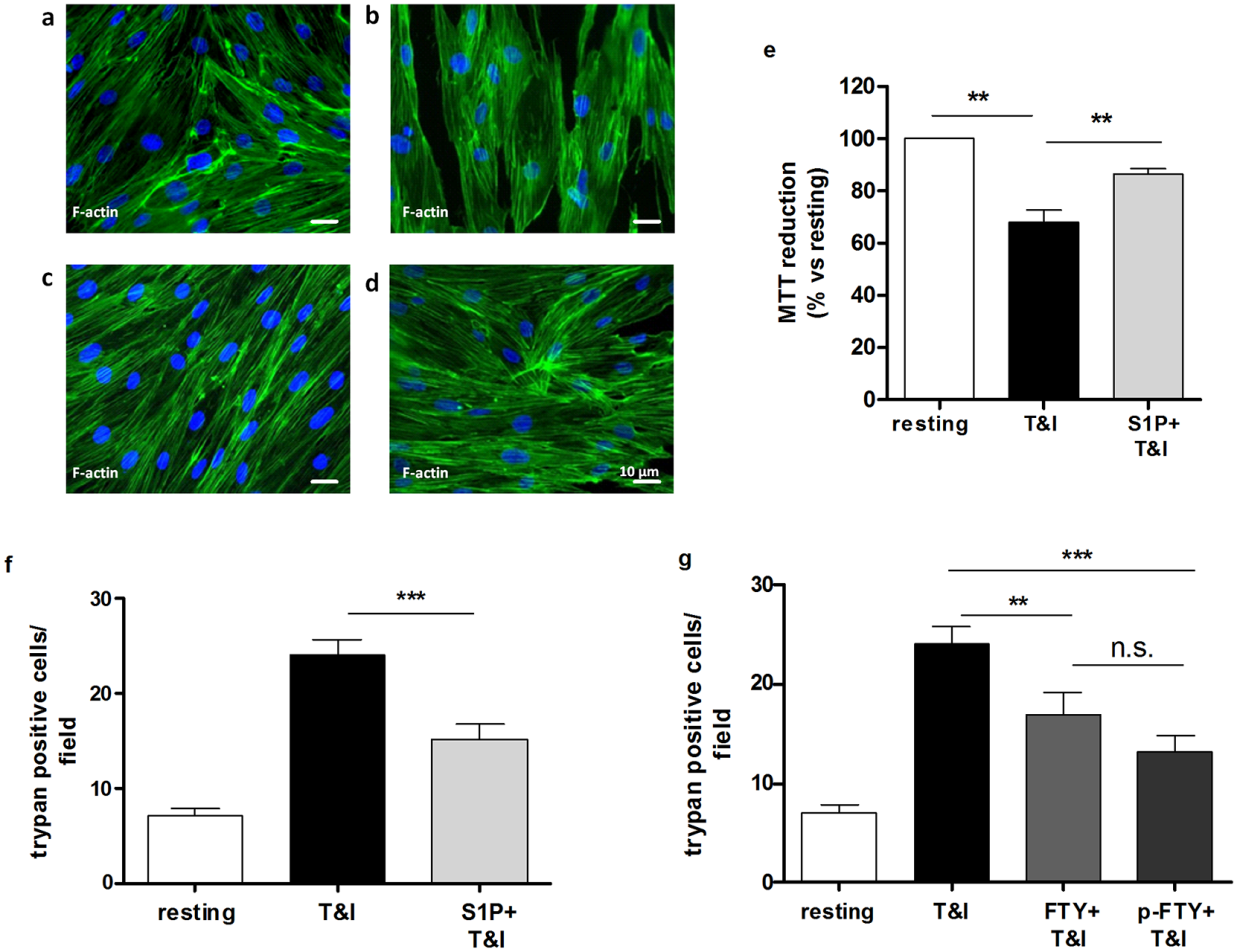
Exposure to T&I and S1P receptor modulators modifies hBMVEC morphology and viability. **a**. Immunostaining for the cytoskeleton component F-actin, shows a confluent monolayer characterized by fusiform morphology and strict cell-to-cell contacts. **b**. Exposure to inflammatory cytokines, TNFα (10 U/ml) and IFNγ (5 U/ml) (T&I), causes changes in hBMVEC morphology as consequence of reduced cell viability. In the presence of S1P (50 nM, **c**) or fingolimod phosphate (pFTY 50 nM, **d**), effects of T&I on hBMVEC morphology are lessened. Scale bar represent 10 μM. **e**. hBMVEC ability to metabolize MTT is reduced as consequence of exposure to T&I for 24 h, while in the presence of S1P (50 nM), this effect is reverted. Values represent absorbance at 570 nm, normalized to resting conditions, in which hBMVEC are considered maximally viable. Data are mean ±S.E.M. of four independent experiments, each run in triplicate. ** p<0.01 vs. T&I, ***p<0.001 vs. T&I. **f**. Trypan blue exclusion demonstrates increased hBMVEC death after 24 h exposure to T&I. S1P (50 nM) co-treatment is conversely able to reduce this effect. **g**. Exposure to T&I in the presence of either FTY (50 nM) or pFTY (50 nM), shows a reduction in hBMVEC death. Data are mean ±S.E.M. of four independent experiments, each run in triplicate, in which three to five fields per well were counted. ** p<0.01 vs T&I, ***p<0.001 vs T&I.

Our previous data show that, when exposed to inflammatory cytokines (TNFα 10 U/ml and IFNγ 5 U/ml, from now on referred as T&I), barrier properties of hBMVEC are reduced, as demonstrated by higher permeability to dextran 10kDa as well as increased protein and mRNA expression of the adhesion molecule ICAM-1 [18]. Further, we observed that cytokines exposure led to endothelial cell loss in our *in vitro* model of the BBB. Endothelial cell compromise is a neuropathological feature of varied clinically relevant inflammatory CNS lesions [22–24]. For this reason we sought to examine hBMVEC viability in our system, providing a useful surrogate for deleterious effects of neuroinflammation on BBB integrity. As shown by staining for F-actin, a structural cytoskeletal protein, hBMVEC constitute a confluent monolayer under resting conditions (Fig 1a): the morphology of the endothelial layer was substantially modified as consequence of T&I exposure (Fig 1b). In particular, hBMVEC appeared hypertrophic, potentially in response to loss of cell-to-cell contacts between adjacent cells. We then investigated whether these morphological changes were correlated to a reduction in cell viability upon cytokine challenge. To this end we performed two different assays, the metabolism of MTT (Fig 1e) and the exclusion of trypan blue dye (Fig 1f). Both assays reported ~30% reduction in cell viability when hBMVEC were exposed to T&I for 24 h (Fig 1e and 1f). Since read-outs for cell viability assessed by MTT and trypan blue exclusion assays were in close agreement, only results of trypan blue assays are subsequently shown.

The exposure of hBMVEC to T&I in the presence of physiological concentrations of S1P (50 nM) reduced the extent of cell death caused by cytokines, as confirmed by the number of trypan blue positive cells (37% reduction; Fig 1f). The beneficial effect on endothelial cell survival mediated by S1P was further reflected by improved integrity of the confluent monolayer (Fig 1c). Notably, fingolimod also modulated endothelial cell viability in this model. After hBMVEC were exposed for 24 h to T&I in the presence of either fingolimod (FTY, 50 nM) or its phosphorylated active form, fingolimod phosphate (pFTY, 50 nM), the extent of cell death was significantly decreased: the morphology of the confluent hBMVEC layer was maintained (Fig 1d) and number of positive trypan blue cells was reduced (Fig 1g). Effects of FTY and pFTY were qualitatively and quantitatively similar suggesting the possibility that pFTY was the active moiety, in line with the general knowledge that fingolimod requires phosphorylation to exert its biological activity [25]. For this reason, accounting for the somewhat larger effect of pFTY, only data demonstrating effects of the phosphorylated drug are shown in subsequent experiments.

### S1P_1_ is the predominant receptor involved in hBMVEC survival

**Fig 2:**
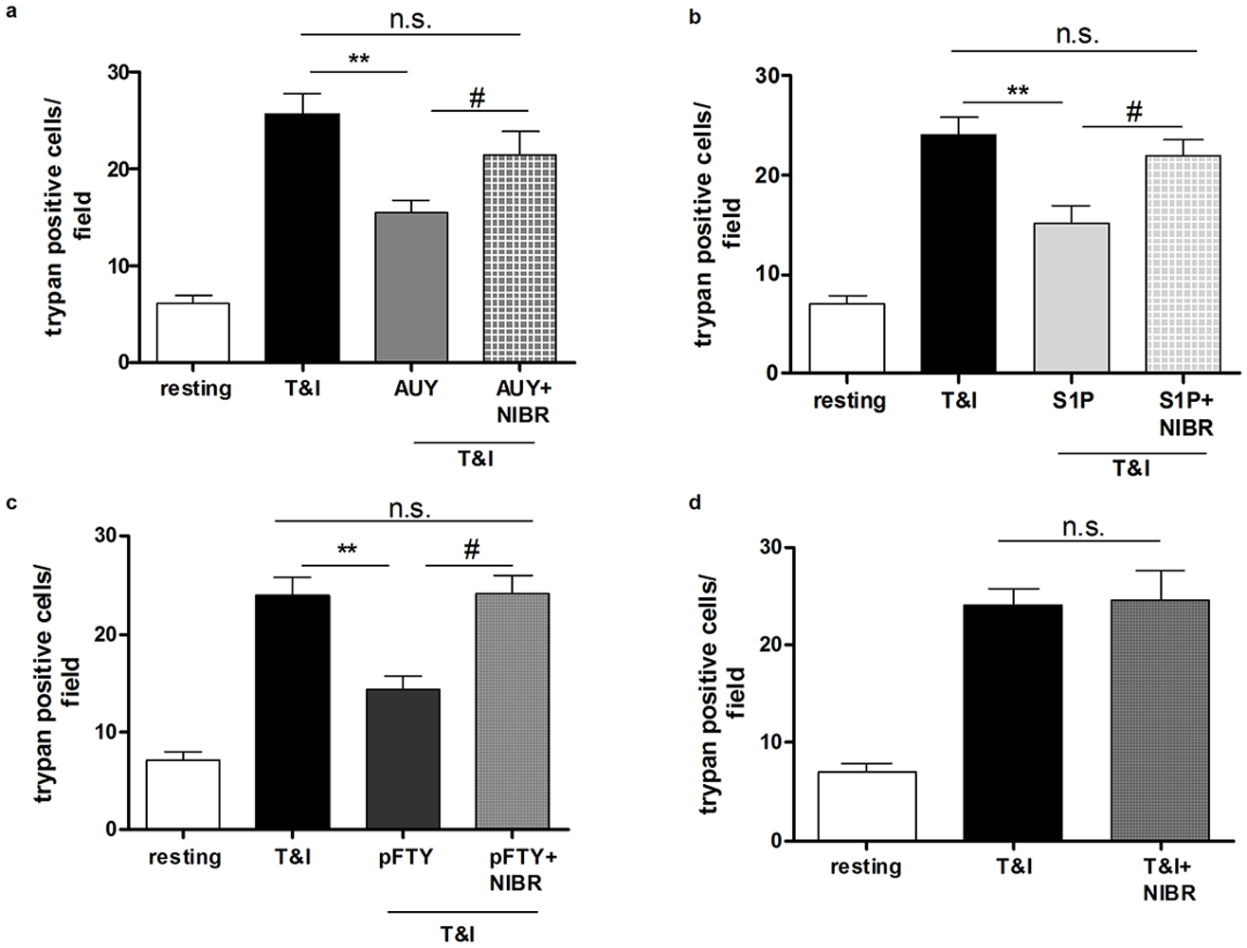
S1P_1_ mediates hBMVEC survival after exposure to cytokines. **a**. The selective S1P_1_ agonist, AUY-954 (AUY, 50 nM) added together with TNFα (10 U/ml) and IFNγ (5 U/ml) (T&I), is able to counteract hBMVEC death induced by cytokines. The presence of S1P_1_ selective antagonist NIBR-0213 (NIBR, 1 μM) prevents this effect entirely. **b. and c**. The principal involvement of S1P_1_ in the protective role of S1P (50 nM) and pFTY (50 nM) is further confirmed by the lack of their effects when used in combination with the antagonist NIBR (1 μM). **d**. The specificity of S1P_1_ antagonism is confirmed by the lack of any effect when NIBR is used in absence of any S1P receptor agonist. Data are the mean ±S.E.M. of four independent experiments, each run in triplicate, in which three to five fields per well were counted. ** p<0.01 vs T&I, # p< 0.05 vs respective treatment in the absence of antagonist.

S1P is active at five distinct receptors (S1P_1-5_), for several of which semi-selective small molecule agonists and antagonists have been developed. Given its anti-inflammatory properties in several settings, we hypothesized that S1P_1_ might be implicated in the effects described above. Indeed using a S1P_1_ selective agonist, AUY-954 (AUY, 50 nM) prevented T&I mediated hBMVEC death (Fig 2a). The involvement of S1P_1_ in hBMVEC protection was further supported using a selective S1P_1_ antagonist NIBR-0213 (NIBR, 1 μM). Accordingly, the protective effects of AUY, as well as of pFTY and S1P was abrogated (Fig 2b and 2c). When hBMVEC were exposed to T&I in the presence of NIBR alone, there was no change in cytokine-mediated toxicity towards hBMVEC (Fig 2d) arguing against any direct effect of NIBR on hBMVEC viability in this model.

### Astrocytes mediate hBMVEC survival in a S1P-mediated manner

When pFTY crosses the BBB, it acts through S1P receptors expressed by cells within the CNS, amongst others [26]. For this reason, the role of S1P modulation on astrocytes was further investigated. First we confirmed that hAST viability was not affected by the exposure to T&I alone or in the presence of S1P (50 nM) or pFTY (50 nM) (data not shown). To investigate if the modulation of S1P receptors on hAST could influence the response of hBMVEC to inflammatory stimuli, hAST were treated for 6 h with T&I either alone or in combination with S1P or pFTY. Fresh medium was added to hAST and kept for 18 h to generate astrocyte conditioned medium (ACM). Then ACM was transferred to hBMVEC cultures, which were subsequently exposed for 24 h to T&I.

**Fig 3:**
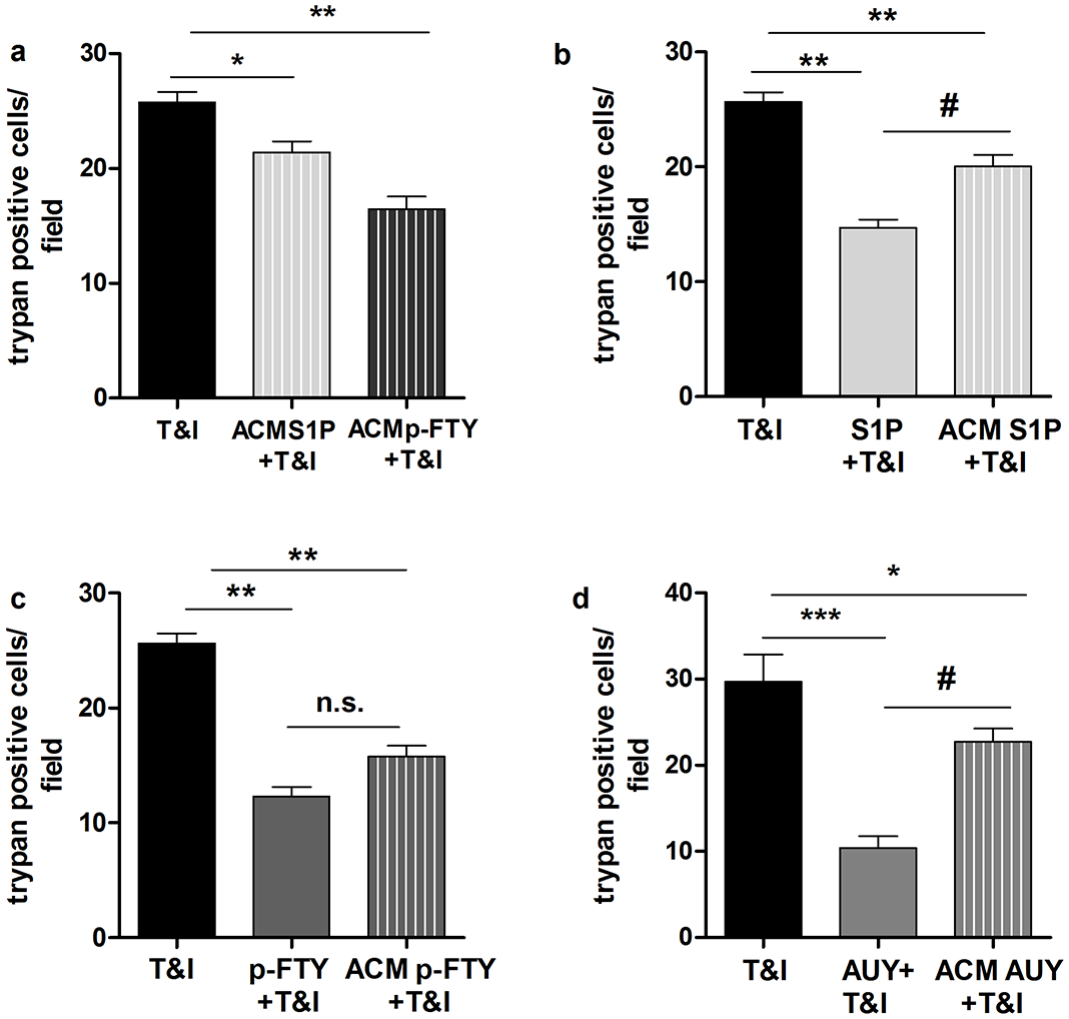
hAST are involved in mediating hBMVEC survival. **a**. Exposure of hBMVECs to T&I in the presence of conditioned medium from hAST (ACM) that were also exposed to T&I in the presence of S1P modulators (S1P 50 nM, pFTY 50 nM), reduces hBMVEC death. **b-d** Direct treatment of hBMVEC with S1P (50 nM, **b**) and AUY (50 nM, **d**) is compared to the effect of ACM exposed to the same compounds. Direct treatment of hBMVEC with S1P or AUY was more effective in preventing hBMVEC death after T&I if compared to treatment with ACM. hBMVEC were equally protected from cell death by T&I challenge when exposed to either pFTY or ACM derived from hAST treated with pFTY (**c**). Data are the mean ±S.E.M. of three independent experiments, each run in triplicate, in which three to five fields per well were counted. *p< 0.05 vs T&I, ** p<0.01 vs T&I, # p<0.05 vs direct treatment on hBMVEC.

Interestingly, ACM affected the response of hBMVEC upon T&I challenge in a beneficial manner. In particular, when hBMVEC were exposed to ACM generated by hAST that were treated with pFTY, the endothelium was less sensitive to T&I induced cell death. The extent of protection mediated by ACM was comparable to direct treatment of hBMVEC with pFTY (Fig 3a–3c). On the contrary, S1P treatment was more efficient when applied directly to hBMVEC (Fig 3b). We then further examined the role of S1P_1_ agonist AUY (50 nM) on hAST. After selective S1P_1_ activation, the ACM released was still able to rescue hBMVEC from T&I challenge. However, as for S1P treatment, the protection mediated by pFTY directly on hBMVEC was more effective (Fig 3d).

### Granulocytes and macrophages colony stimulating factor (GM-CSF) is one of the factors released by hAST able to increase hBMVEC viability after T&I challenge

**Fig 4:**
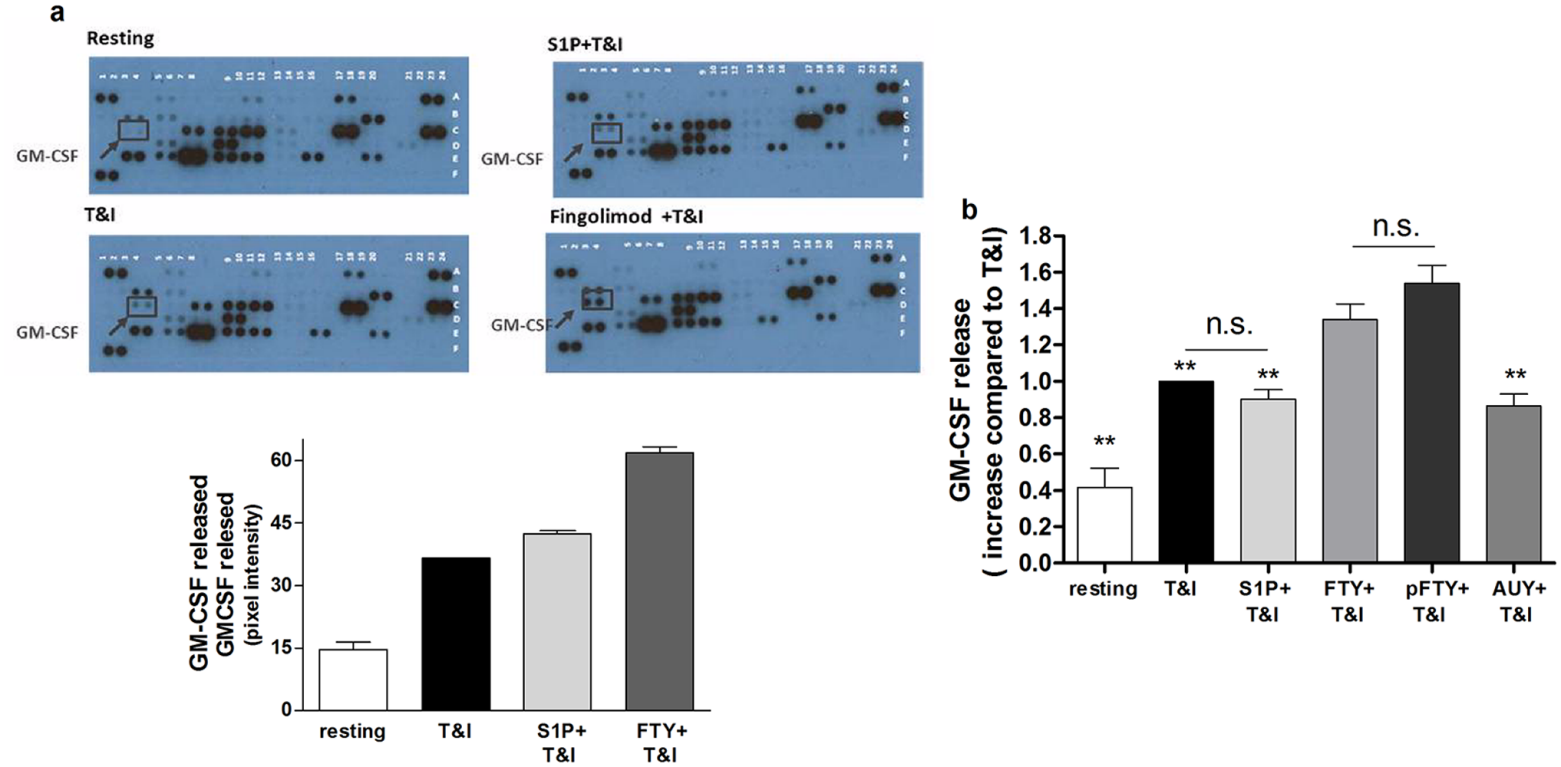
hAST release GM-CSF after S1P modulation. **a**. hAST are exposed to T&I (10 U/ml, 5 U/ml) either alone or in combination with S1P (50 nM) or FTY (50 nM) for 6 h then washed and kept in fresh media for 18 h. The supernatant, was collected and released factors were analyzed by a cytokine/growth factor array (R&D Systems). The upper panel shows blots to identify factors present in the ACM after stimulation as indicated; the lower panel shows quantification of the released GM-CSF (pixel intensity). **b**. ELISA evaluation of the released GM-CSF after T&I exposure either alone or in the presence of S1P (50 nM), FTY (50 nM) pFTY (50 nM) and AUY (50 nM) (fold increase against T&I is shown in the graph).** p<0.05 vs pFTY+T&I.

The effects described above were consequences of protein based factors released in the medium by hAST after their stimulation by cytokines, as heat-inactivated ACM could not prevent hBMVEC from cytokine-induced cell death (data not shown). We then aimed to identify such soluble factors by performing a limited cytokine array with media released by hAST treated with T&I either alone or in the presence of FTY or S1P. Several factors involved in the angiogenic pathway were detected, and for some of those we also observed variations during treatments (Table 1). The factor showing the highest concentration over baseline was granulocytes and macrophages colony stimulating factor (GM-CSF, Fig 4a and Table 1). Next, the release of GM-CSF by hAST after S1P modulation was confirmed and quantified using an ELISA assay. hAST secretion of GM-CSF after T&I activation was not modified by S1P or AUY, but was markedly increased after FTY and pFTY stimulation. Both FTY and its phosphorylated form induced a comparable release of GM-CSF (Table 2 and Fig 4b). hBMVEC did not produce significant amounts of GM-CSF under any conditions of treatments (Table 2).

**Table 1:** Summary of the principal factors released by hAST into the media after T&I exposure either alone or in combination with S1P or FTY as determined by a limited Proteome Profiler Human Angiogenesis Array (R&D Systems). For each factor, densitometric analysis of band intensity was carried out with the Image J Software developed by NIH. Values are indicated as percent variation over resting condition.

	RESTING	T&I	S1P+T&I	FTY+T&I		RESTING	T&I	S1P+T&I	FTY+T&I
Activin A	100	87,2	95,3	87	IL-8	100	99,9	94,5	88
Angiopoietin-1	100	47,5	54,2	67,2	CCL2	100	104,7	108	105
Amphiregulin	100	84,3	87,4	75,8	MMP9	100	78,1	83,9	83,7
Coagulation factor III	100	0	146,6	147,1	Pentraxin3	100	89,7	101,1	106,9
CXCL16	100	182	150,9	196,5	PDGF-AA	100	0	0	0
DPPIV	100	54,1	93,6	76,2	PIGF	100	91,6	156	0
Endoglin	100	0	0	0	Serpin E1	100	92,8	102,1	101,6
FGF-basic (2)	100	88,9	112,2	106,2	Serpin F1	100	80	83,6	81
GM-CSF	100	347	395,7	956,5	TIMP-1	100	90,5	96	95,6
HGF	100	84,7	93,5	92,8	TIMP-4	100	86,2	98,5	98,9
IGF-BP1	100	101,8	109,8	116,2	Thrombospondin-1	100	81,6	101,7	109,4
IGF-BP2	100	95,3	92,8	101,2	uPA	100	76,3	87,4	60,8
IGF-BP3	100	0	85,5	68	VEGF	100	92,1	91	103

**Table 2:** Evaluation of GM-CSF release by ELISA. Values report the quantification of GM-CSF released by hAST and hBMVEC, respectively, after T&I exposure either alone or in the presence of S1P (50 nM), FTY (50 nM), pFTY (50 nM), and AUY (50 nM).

	Astrocytes	Endothelial cells
**Resting**	30.9 pg/ml	3.2 pg/ml
**T&I**	86.1 pg/ml	7.7 pg/ml
**T&I+S1P**	82.3 pg/ml	7.8 pg/ml
**T&I+FTY**	144.1 pg/ml	9.7 pg/ml
**T&I+pFTY**	150.2 pg/ml	8.7 pg/ml
**T&I+AUY**	84.5 pg/ml	8.4 pg/ml

### GM-CSF rescues hBMVEC from T&I-mediated cell death

**Fig 5:**
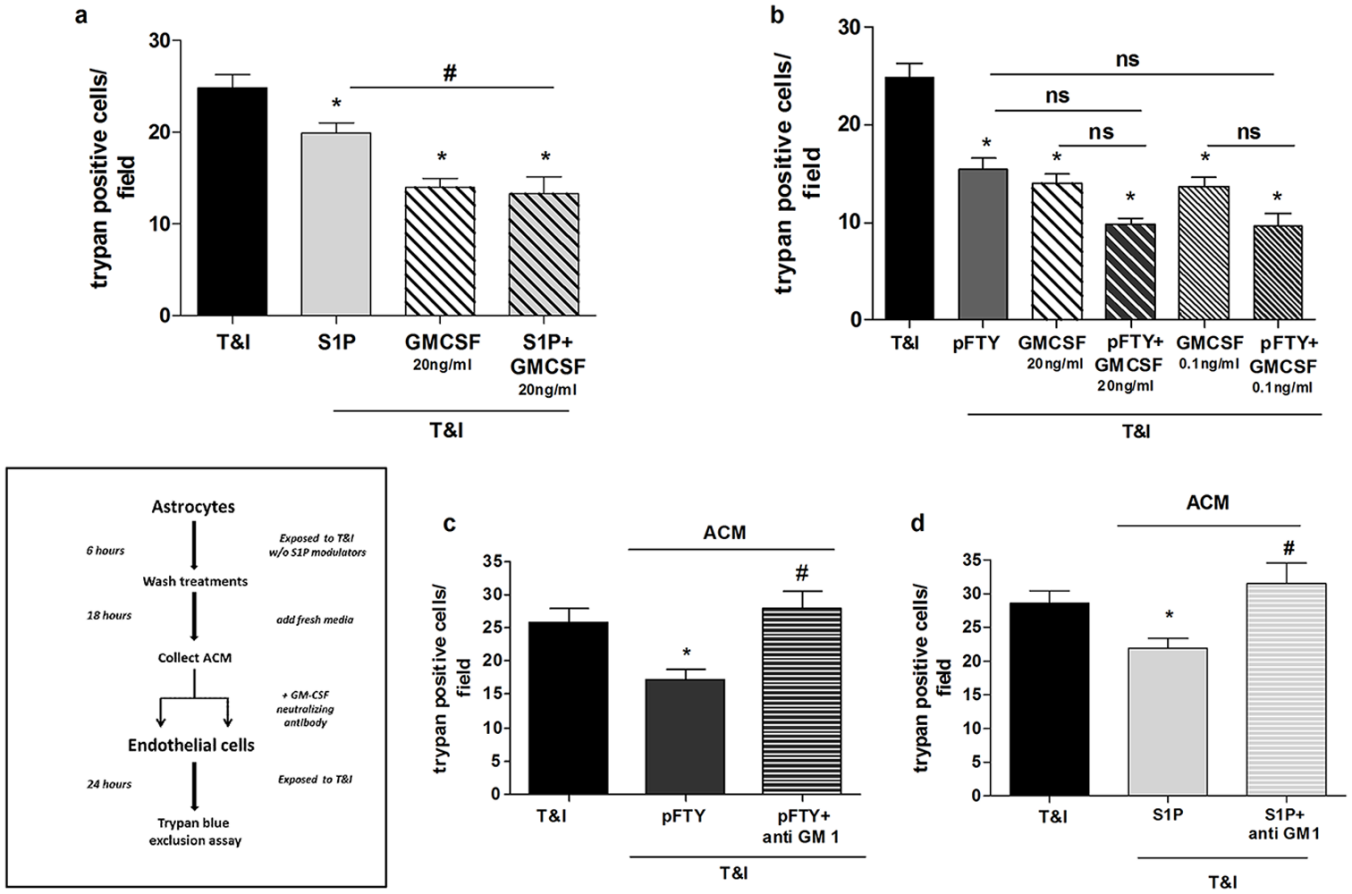
GM-CSF reduces hBMVEC death induced by cytokines. **a**. hBMVEC were exposed to T&I in the presence of GM-CSF (20 ng/ml) either alone or in combination with S1P (50 nM). The protection induced by the growth factor was significantly increased over S1P alone, whereas the combination of the two induced an effect comparable to GM-CSF alone. *p< 0.05 vs T&I, # p<0.05 T&I+S1P. **b** Exposure to GM-CSF (20 ng/ml or 0.1 ng/ml) was able to counteract T&I induced hBMVEC death. The effect of the growth factor was comparable to the effect of pFTY (50 nM). When the two compounds were used in combination, their effect was less than additive. **c-d**. When astrocytes conditioned medium (ACM), derived from hAST treated with S1P (c) or pFTY (d), was transferred to hBMVECs in combination with a neutralizing antibody against GM-CSF (1 μg/ml), the protective effect of GM-CSF was abolished, as demonstrated by increased hBMVEC death. Data are the mean ±S.E.M. of three independent experiments, each run in triplicate, in which three to five fields per well were counted.*p< 0.05 vs T&I, # p<0.05 vs the respective treatment in absence of the neutralizing antibody.

We next assessed the direct effects of GM-CSF on hBMVEC viability. As expected, T&I-induced cell death was reduced in the presence of GM-CSF (20 ng/ml, Fig 5a and 5b). The extent of hBMVEC protection mediated by the growth factor was compared to the one mediated by either S1P or pFTY (Fig 5a and 5b, respectively): GM-CSF was more efficient in exerting hBMVEC protection when compared to S1P; when the two agents were added together there was not enhancement in hBMVEC survival (Fig 5a). Conversely, protection mediated by GM-CSF was comparable to pFTY treatment (Fig 5b); when added together, pFTY and GM-CSF exerted a sub-additive effect, suggesting action through overlapping pathways (Fig 5b). The protective effect of GM-CSF was also seen when used at a concentration as low as 0.1 ng/ml (equal to the amount that hAST released after pFTY treatment) (Fig 5b). To address whether GM-CSF in ACM is necessary to rescue hBMVEC from T&I-induced cell death, we added a neutralizing antibody against GM-CSF (anti GM, 1 μg/ml R&D Systems) to ACM. Indeed GM-CSF antibodies rendered ACM that was generated in the presence of pFTY, inert for mediating protection of hBMVEC from T&I-induced toxicity (Fig 5c). A similar effect was observed when the GM-CSF neutralizing antibody was combined with ACM generated by hAST after S1P treatment (Fig 5d).

**Fig 6:**
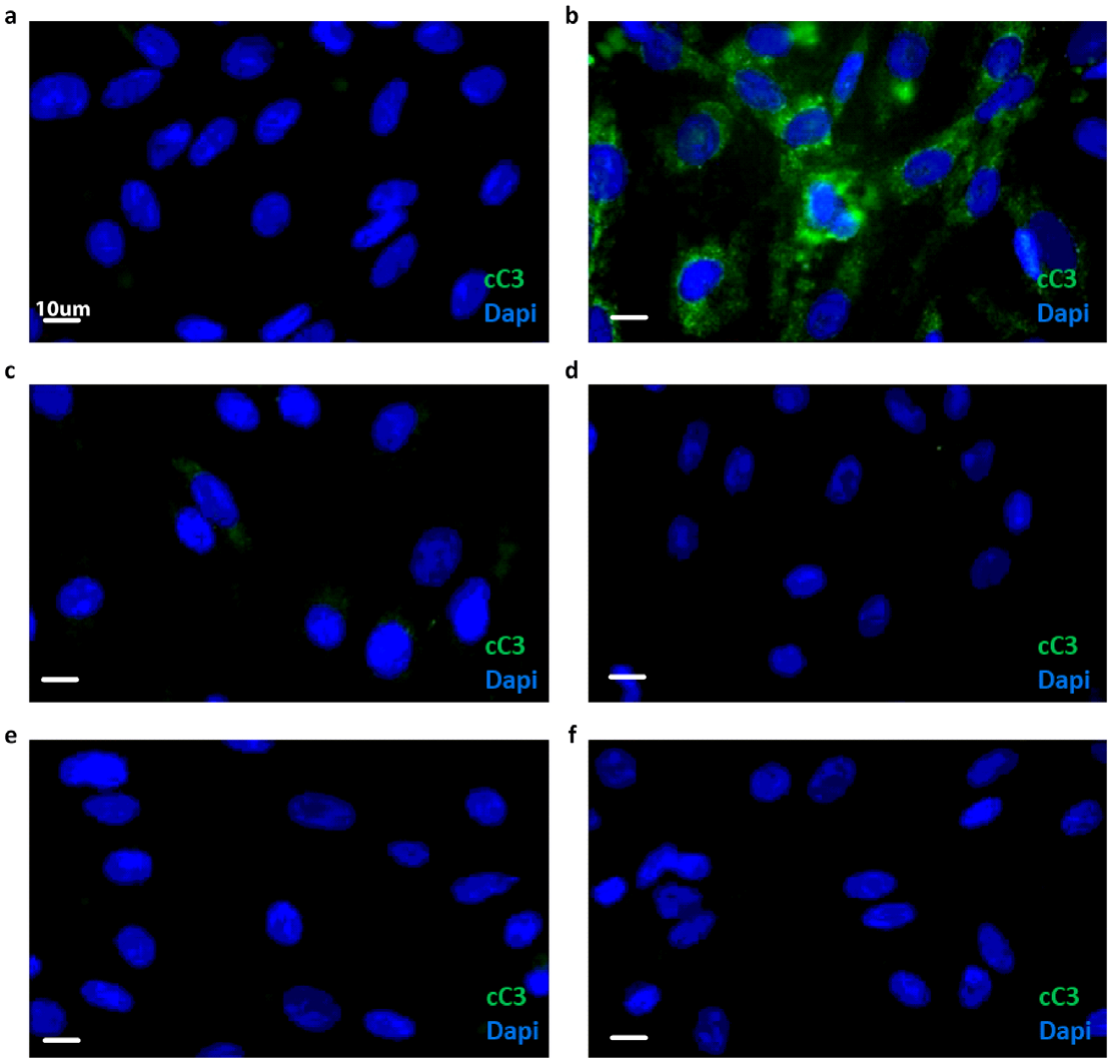
The decrease in hBMVEC death is associated with reduced activation of Caspase-3. hBMVEC were treated as indicated, and then fixed and immunostained for the cleaved active form of Caspase-3 (cC3). Resting hBMVEC, i.e. not exposed to inflammatory cytokines, did not express cC3 **(a)**. When hBMVEC were exposed to T&I (10 U/ml and 5 U/ml) for 3 h alone (**b**) or in combination with S1P (50 nM, **c**) pFTY (50 nM, **d**), AUY (50 nM, **e**) or GM-CSF (100 pg/ml, **f**), only T&I-activated hBMVEC showed presence of cC3, whereas. all treatments reverted the effect induced by T&I. Images are representative of three independent experiments. Scale bar represents 10 μm.

To examine whether GM-CSF mediates hBMVEC survival by rescuing them from undergoing apoptosis, we added the growth factor directly to hBMVECs in the presence of T&I, and performed immunostaining for cleaved Caspase-3 (cC3), a marker for apoptosis, which was undetectable in resting hBMVEC, as expected (Fig 6a). However T&I induced a massive enhancement in cC3 immunoreactivity (Fig 6b), which was abolished by GM-CSF treatment (Fig 6f). The same protective effect was observed when hBMVECs were exposed to S1P, pFTY and AUY (Fig 6c and 6e).

### S1P receptor modulation reduces transmigration of PBMCs in an *in vitro* BBB model incorporating flow

**Fig 7:**
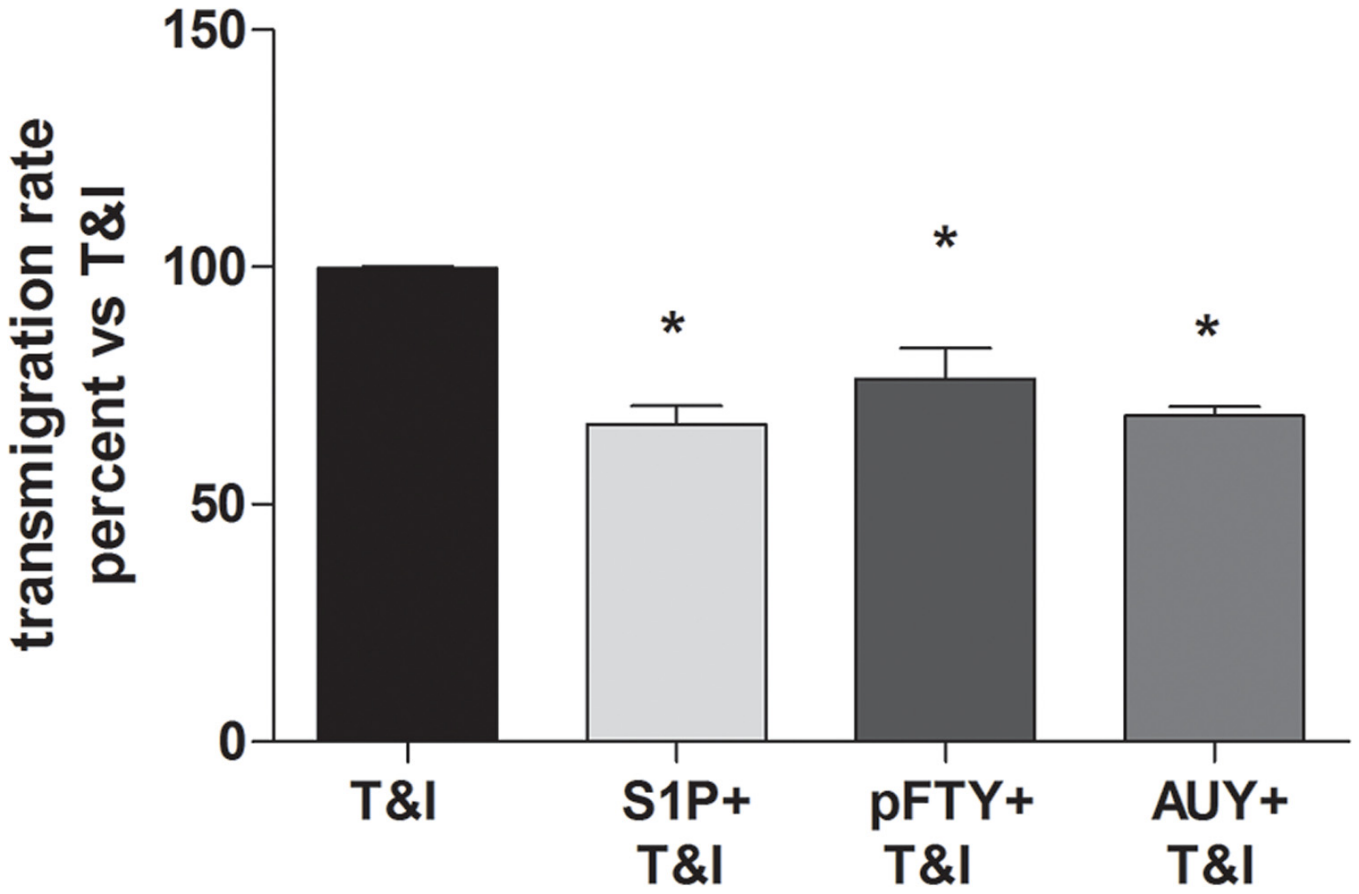
S1P modulation reduces transmigration of PBMCs across an *in vitro* BBB model under shear stress. When hBMVEC/hAST co-culture was exposed to T&I in the presence of S1P (50 nM), pFTY (50 nM), or AUY (50 nM), the ability of PBMCs to migrate through the barrier is reduced if compared to the condition in which the barrier is exposed only to cytokines. Data are mean of 2 independent experiments and are expressed as percent reduction in PBMC migration after 1 hour compared to T&I. *p< 0.05 vs T&I.

Previously, we demonstrated in our *in vitro* BBB system comprising hBMVEC/hAST co-culture that cytokines exposure of hBMVEC promotes transmigration of PBMCs under flow [18]. To evaluate whether treatment with S1P or pFTY could modulate this effect in the settings of inflammation, we performed an *in vitro* PBMC transmigration assay. After plating hAST and hBMVEC on opposite sides of collagen-coated membranes [18], cells were activated with T&I (10 U/ml & 5 U/ml) either alone or in the presence of S1P (50 nM), pFTY (50 nM), or AUY (50 nM). Numbers of migrated cells after one hour were quantified. Treatment with S1P, pFTY or AUY significantly reduced numbers of migrated PBMCs across cytokine-activated hBMVEC/hAST co-culture membranes under conditions of physiological shear stress (Fig 7).

## Discussion

### Acting through S1P1, fingolimod reduces damage of BBB endothelial cells in inflammatory conditions

In the present study, we analyzed the effects of S1P signaling on the integrity of an *in vitro* BBB endothelium and on the maintenance of BBB properties under cytokine stress. To this end, we performed assays in which we either assessed direct effects on endothelial cells or indirect effects through the induction of trophic factors released by astrocytes. S1P receptor signaling on the endothelium has been examined in different models, both *in vitro* and *in vivo*, and the bioactive sphingolipid S1P has been associated with a number of different effects on endothelial cells. As one example S1P modulates vascular barrier properties, either enhancing or reducing endothelial cell cytoskeletal stability and tight junction and adherens junction rearrangement [27–31]. This dual response exerted at the endothelium results from the modulation of different S1P receptors [32]. Endothelial cells express at least three types of S1P receptors (S1P_1-3_), which are required to develop and maintain a functional vascular system [33]. Although S1P_1-3_ cooperate in the maintenance of endothelial cell integrity, individually they play distinct roles with regard to cytoskeletal contractility and cell-to-cell contacts, altogether modifying vascular permeability [34, 35]. S1P_1_ signaling has been implicated in reducing plasma solute permeability, as well as restricting leukocyte infiltration in inflamed areas [36]. S1P_2_ conversely increases vascular permeability due to modification of cytoskeletal forces [30, 37] and enhances the expression of inflammatory markers inducing leukocyte transmigration [36, 38]. A proper equilibrium in the signaling between S1P_1_ and S1P_2_ is essential for the maintenance of a competent endothelial barrier at the BBB, further regulating leukocyte infiltration into the CNS [39] [31, 35]. Our results suggest that S1P receptor activity can also modify the response of endothelial cells to cytokines as monitored by indicators of viability. Although loss of endothelial cells cannot be considered a typical hallmark in the pathogenesis of MS, a fully competent endothelial barrier is required to maintain BBB properties, including restricted paracellular permeability and limited migration of PBMCs into the CNS. Notably, in diverse neuroinflammatory conditions, functioning and viability of endothelial cells are compromised [23, 24, 40, 41].

In our *in vitro* BBB model the exposure to TNFα and IFNγ, cytokines often increased in inflammatory conditions, can lead to endothelial cell death. Treatment with S1P receptor modulators significantly improved endothelial cell viability. This finding is in concordance with the recent demonstration that S1P treatment of human umbilical vein endothelial cells (HUVECs) reduced apoptotic death after serum withdrawn [32]. As already discussed, several S1P receptors are expressed on the endothelium, and they all contribute differently to endothelial barrier properties. We here provide evidence that S1P_1_ is the receptor mostly involved in the protective effects described. Our attention to S1P_1_ is based on the knowledge that the immunomodulator fingolimod acts on all S1P receptor but S1P_2_, with a higher affinity for the type 1 receptor [6]. Throughout our study, we tested a physiological concentration for free S1P (the active available plasma concentration of S1P is ~ 50 nM [42]), that mainly signals through S1P_1_, whose affinity for S1P is the highest. Preliminary data, not shown here, indicate that in our *in vitro* model of the BBB, S1P concentrations above 200 nM cause endothelial barrier breakdown, most likely as result of S1P2 signaling. These data are in concordance with recent findings in which the expression and the activity of S1P_2_ are associated with increased BBB permeability and more severe signs in experimental autoimmune encephalomyelitis (EAE), an animal model of neuroinflammation in MS [37]. We further examined the specific involvement of S1P_1_ using a selective agonist, AUY-954, whose activity at S1P_1_ reduced EAE severity *in vivo* [43]. Our data provide evidence that enhanced endothelial cell viability after treatment with fingolimod, or its phosphorylated active form, is consistent with the effects mediated by a S1P_1_ selective agonist or the biological ligand, S1P. In our system the prodrug fingolimod and its phosphorylated form, the one that exerts the biological activity *in vivo* [25], show superimposable effects, indicating that endothelial cells and astrocytes are able to phosphorylate, thus activating, the prodrug.

Fingolimod's efficacy as a treatment for MS has been attributed to its functional antagonism, due to its ability to temporarily activate S1P_1_ and to cause its internalization and degradation. The reduced expression of S1P_1_ on T cells during prolonged fingolimod treatment causes a drop in their egress from lymph nodes [44]. Conversely, the bioactive molecule S1P, after an initial internalization, allows the quick relocalization of S1P_1_ back to the membrane surface [45, 46]. Here, we report the effects of the prodrug fingolimod and its active form, associated with their functioning as agonist at S1P_1_. Strikingly the drug exerts effects that are more potent than those of S1P or AUY-954 for maintenance of endothelial cell viability. The protective effect was lost when agonists were administered in the presence of NIBR-0213, a selective S1P_1_ antagonist [47]. This finding further confirms the principal involvement of S1P_1_ in the rescue of endothelial cells upon cytokine stress.

### When acting behind the BBB on astrocytes, fingolimod ameliorates endothelial viability in the presence of inflammatory cytokines

Endothelial cells are the primary cellular constituents of the BBB, but a complex network of other cell types are present within the neurovascular unit to support and regulate endothelial barrier function. Interactions are implemented by direct physical contact or through soluble factors.

In adulthood, astrocytes are associated with maintenance of the BBB properties [48]. Inflammatory conditions induce reactive astrogliosis, accompanied by the release of soluble mediators that affect neurons and oligodendrocytes as well as endothelial cells [49, 50]. But in the same context astrocytes can also release anti-inflammatory mediators such as retinoic acid, that support BBB integrity [51]. During MS, the expression of S1P_1_, S1P_3_ and S1P_5_ on reactive astrocytes is increased [15, 52] [53], prompting several groups to investigate the effects that S1P modulation could exert in inflammatory conditions. Astrocytes exposure *in vitro* to fingolimod reduces the release of CCL2 induced by TNFα [53], suggesting that fingolimod acts through S1P_1_ or S1P_3_, to dampen the astrocytic inflammatory responses. Further, in EAE models, the selective deletion of S1P_1_ from astrocytes rendered fingolimod ineffective. Consequently, pathology could no longer be ameliorated by fingolimod. It is of note, however, that untreated-control EAE scores in these mice were so low as to leave very little window to demonstrate further benefit from fingolimod treatment [54].

Here we show that S1P receptor agonists stimulate astrocytes to produce factors that limit endothelial cell death after exposure to TNFα and IFNγ. Some of the factors that were modified by the treatments examined are appealing, due to the fact that they are mostly involved in proliferation and morphological differentiation of endothelial cells. This is the case, for example, for CXCL16. The chemokine is released by astrocytes when exposed to T&I [55], unaffected by either fingolimod or S1P. It has been reported that *in vitro*, CXCL16 stimulates neoangiogenesis in HUVECs [56]. In our case the effects induced by ACM on hBMVEC cannot be ascribed to CXCL16, since brain endothelial cells do not express its receptor, CXCR6 [57]. Other factors, like placental growth factor (PlGF), able to promote angiogenesis as well [20], show opposite results upon S1P and FTY treatment, so it is rather unlikely that they mediate the effects we observed. Among other factors that we identified in ACM, GM-CSF accounts, at least in part, for the protective effects herein described. GM-CSF, a key determinant for myeloid lineage differentiation, has been associated with inflammatory responses in several disease models [58]. In particular, Th1- and Th17-derived GM-CSF triggers recruitment of inflammatory macrophages to the CNS during EAE [59, 60]. Conversely, GM-CSF has been reported to mediate maintenance of intestinal tolerance and prevention of inflammatory bowel disease [61]. Moreover in the CNS, levels of GM-CSF and its receptors are increased after ischemic insult, associated with reduced apoptotic neuronal loss, suggesting a neuroprotective role for the cytokine [62]. Our study now demonstrates that astrocyte-derived GM-CSF also protects endothelial cells from undergoing apoptosis induced by the exposure to inflammatory cytokines. Supportive evidence for an anti-inflammatory role of GM-CSF comes from an *in vitro* study that reported a reduced expression of adhesion molecules ICAM-1 and VCAM-1 when endothelial cells were treated with GM-CSF under inflammatory conditions [63]. These findings further emphasize the importance of the factor released by astrocytes in the context of maintaining BBB function in pathological conditions like MS. Finally, the reduced transmigration of PBMCs across our cytokine-activated *in vitro* BBB model under shear stress may serve as functional proof of concept, demonstrating that S1P modulation results in ameliorated barrier functions, dampening the inflammatory response of the BBB endothelium under pathological conditions.

## Conclusions

Our data suggest that S1P agonism enhances BBB properties due to its activity on both endothelial cells and astrocytes: on endothelial cells, acting through S1P_1_, S1P increases their ability to survive cytokine challenge, while acting on astrocytes, fingolimod induces GM-CSF release that further increases BBB stability. One limitation of this research inheres in the effects of acute treatment with S1P agonists. Clinical benefit of fingolimod treatment is attributed to functional antagonism of S1P_1_ on T cells and was recapitulated using the S1P_1_ selective antagonist, NIBR-0213 [47]. Moreover, chronic use of S1P_1_ selective antagonists in animal models led to pulmonary vascular leakage, due to the inhibition of S1P_1_ activity on endothelial cells [64]. Further investigations will be required to determine whether long-term fingolimod treatment maintains agonistic activity towards astrocytes and endothelial cells at the BBB. If confirmed in *in vivo* models, the effects that fingolimod mediates in both endothelium and astrocytes, reinforcing BBB integrity and function, may be added to its mechanisms of action in the treatment of neuroinflammatory disease.
